# Psychotherapy for depression in college students: A protocol for systematic review and network meta-analysis

**DOI:** 10.1097/MD.0000000000022344

**Published:** 2020-09-25

**Authors:** Xiu Zhang, Ming-Ming Niu, Pei-Fen Ma, Li Du, Lin Wan

**Affiliations:** a Department of Orthopedics, Second Hospital of Lanzhou University; b Evidence-Based Nursing Center, School of Nursing, Lanzhou University; c Department of Nursing, Second Hospital of Lanzhou University; d School of Nursing, Lanzhou University; e The Third People's Hospital of Lanzhou city, Lanzhou, China.

**Keywords:** college students, depression, network meta-analysis, psychotherapy

## Abstract

**Background:**

Depression is a disease with a high incidence and easy to relapse. It not only affects the work and life of patients, but also brings a heavy economic burden. University is the peak of depression, and the prevalence of depression among college students is much higher than that of ordinary people. The purpose of this research is to evaluate depression symptoms, life satisfaction, self-confidence, substance use, social adjustment, and dropout rates of the use of psychological intervention for college students.

**Methods:**

We will identify relevant trials from systematic searches in the following electronic databases: PubMed, Embase, Web of Science and The Cochrane Library. We will also search Clinical Trials.gov, the WHO International Clinical Trials Registry Platform for unpublished data. Additional relevant studies will be searched through search engines (such as Google), and references included in the literature will be tracked. All relevant randomized controlled trials (RCTs) will be included. There are no date restrictions. Use Cochrane Collaboration's Risk of bias tool to conduct risk of bias analysis. Use the Grades of Recommendation, Assessment, Development, and Evaluation to assess the quality of evidence. All statistical analysis will be performed using Stata (V.15.0.) and Review Manager (V.5.2.0).

**Results:**

A total of 6238 records were obtained by searching the database and 27 records were obtained by other sources. After removing duplicate records, there are 4225 records remaining. We excluded 3945 records through abstract and title, leaving 280 full-text articles.

**Conclusion:**

This will be the first study to compare the effects of different psychological treatments on depression in college students. We hope that this study will guide clinical decision-making of psychotherapy to better treat depression in college students.

**Protocol Registration:**

INPLASY202070134.

## 1 Introduction

Depression is a common mental health disorder, which is mainly manifested by significant and lasting depression, slow thinking, sleep disturbance, loss of appetite, etc. In severe cases, suicide attempts or behaviors may occur.^[1]^ Each episode of depression lasts at least two weeks. In severe cases, it may last for several years. This has a serious impact on work and life, and has caused a heavy financial burden. According to the World Health Organization, more than 350 million people worldwide suffer from depression.^[2]^ The current incidence of depression in China is 6.1%.^[3]^ By 2020, depression may become the second largest disease after heart disease.^[4]^ And depression has become the main reason for people's loss of social function and ranks third in the global burden of disease.^[5]^ Studies have shown that in the United States alone, the annual cost exceeds $43.7 billion.^[6,7]^ College students are faced with the pressure from interpersonal communication, arduous learning tasks and adaptation to the new environment and lifestyle, which makes them prone to produce strong psychological conflicts and lead to depression.^[8]^ Therefore, compared with their peers, college students have a higher risk of depression.^[9]^

At present, the treatment of depression is mainly divided into medication and psychotherapy. Drug therapy mainly includes selective serotonin reuptake inhibitors (SSRIs), tricyclic antidepressants (TCAs), serotonin norepinephrine reuptake inhibitors (SNRIs), etc.^[10]^ Psychotherapy is to establish a relationship with the patient through a structured and purposeful connection and use a series of specific techniques to improve the patient's mental state.^[11]^ It plays an important role in the treatment of depression. At present, the common psychotherapy in clinical treatment methods include cognitive behavior therapy, group psychotherapy, interpersonal behavior therapy, mindfulness therapy, etc. Previous studies showed that there are few systematic reviews and meta-analysis of depression in college students. However, the relevant evidence for the effectiveness of psychotherapy is still unclear, and there is no evidence to directly compare different psychological interventions. Therefore, this field urgently needs a Bayesian network meta-analysis (NMA) method that combines direct evidence with indirect evidence from multiple treatment comparisons to estimate the correlation between all treatments.^[12]^ In this study, we will conduct a systematic review and NMA to evaluate depression symptoms, life satisfaction, self-confidence, substance use, social adjustment, and dropout rates of the use of psychotherapy for college students.

## 2 Methods

### 2.1 Eligibility criteria

#### 2.1.1 Type of study

We will include all relevant randomized controlled trials (RCTs) including crossover trials. There are no language restrictions.

#### 2.1.2 Type of patient

The patients we will include are college students diagnosed with depression according to any diagnostic criteria, such as Diagnostic and Statistical Manual of Mental Disorders (DSM)-III,^[13]^ DSM-IV,^[14]^ and International Classification of Diseases, 10th Revision (ICD-10).^[15]^ Studies in which participants have a diagnosis of bipolar disorder, psychotic depression will be excluded. In addition, studies where participants are not clearly diagnosed with depression will also be excluded.

#### 2.1.3 Type of interventions

We will include RCTs comparing one psychological intervention with another control conditions for depression in college students. For psychotherapy, mindfulness therapy, cognitive-behavioral therapy (CBT), meditation therapy, comprehensive self-control training (CSCT),^[16]^ acceptance and commitment therapy (ACT), ^[17]^ and behavioral activation (BA) will be included. There will be no limit to the treatment session. In terms of control conditions, waiting-list control (WLC),^[18]^ non-treatment control, physical exercise, bibliotherapy,^[19]^ treatment as usual (TAU) will be included.

#### 2.1.4 Type of outcomes

Primary outcome

Depression symptoms that mean the change in severity of depression from baseline to end point which is measured by the depression scale, such as Beck Depression Inventory (BDI),^[20]^ The Center for Epidemiologic Studies Depression Scale (CESD-R),^[21]^ Hamilton Rating Scale for Depression (HRSD).^[22]^

Second outcomes

1. self-confidence, life satisfaction was assessed using visual rating scale
2. social adjustment was assessed using the Social Adaptation Self Evaluation Scale (SASS) ^[23]^ and the Social Adjustment Scale-Self Report for Youth.^[24]^
3. substance use was measured with 10 items to assess the use of eight substances, quantity per drinking and smoking day.^[25]^
4. Dropout rates from the beginning of the study to the end of the intervention.

### 2.2 Data source

We will identify relevant trials from systematic searches in the following electronic databases: PubMed, Embase, Web of Science and The Cochrane Library. We will also search Clinical Trials.gov, the WHO International Clinical Trials Registry Platform for unpublished data. The search terms will include “depression”, “depressive disorder”, “students”, “university student”, “college student”. Additional relevant studies will be searched through search engines (such as Google), and references included in the literature will be tracked. There is no date restriction. Detail of search strategy of PubMed is shown in Table 1 as well as detail of search strategy of Embase is shown in Table 2.

**Table 1:**
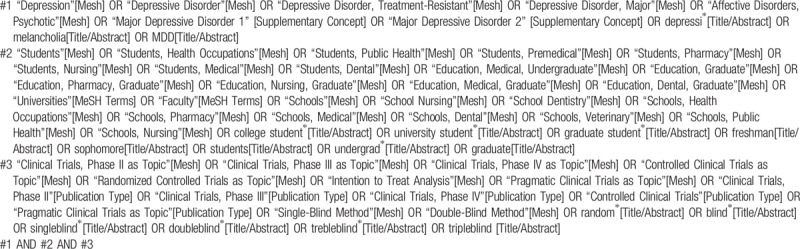
Searching strategy in PubMed.

**Table 2:**
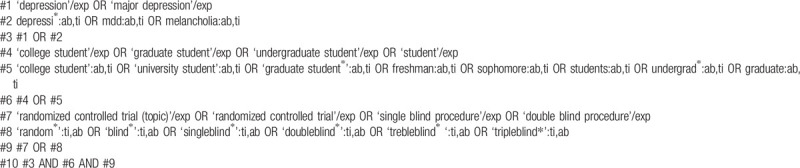
Searching strategy in Embase.

### 2.3 Study selection

All records identified in the databases will be collected in the reference management software EndNote X8 for data screening. Two (MMN and PFM) reviewers will use data extraction tables to extract data from the original report independently, including research characteristics (such author information, publication year, journal and country), patient characteristics, intervention and outcome. Any disagreements will be resolved by the third member of our review team.

### 2.4 Risk of bias analysis

According to Cochrane Collaboration's Risk of bias tool, we will assess risk of bias as ‘low risk’, ‘unclear risk’ or ‘high risk’.^[26]^ The following items will be evaluated: sequence generation, allocation concealment, blinding of participants and personnel, blinding of outcome assessors, incomplete outcome data and selective outcome reporting and other sources of bias.^[27]^ The evaluation will be conducted by two independent raters (PFM and LD). Any disagreements will be resolved by a third review author.

### 2.5 Statistical analysis

#### 2.5.1 Pairwise meta-analysis

We will use Review Manager (V.5.2.0) to perform traditional pairwise meta-analysis. Dichotomous data will be expressed as relative risk (RR) with 95% confidence interval (CI), and continuous outcomes will be expressed as standard mean difference (SMD) with 95% CI.^[28]^

#### 2.5.2 Network meta-analysis

To simultaneously assess the comparative effects of more than 2 psychotherapy, an NMA will be performed. An NMA synthesizes direct and indirect comparisons over an entire network of psychotherapy, allowing for all available evidence to be considered in one analysis. Based on the network development process as outlined above, the outcome variable for the NMA is the standardized mean change in the DSST (measured using Hedge's G) from baseline to end of study. The standardization is based on the pooled (across treatment arms within study) estimate of the SDs. The NMA will be carried out using a frequentist's approach, and a 2-way ANOVA model is used. As the residual variances between treatment groups are known, it is possible for random effect estimates to be produced, which account for the between-trial heterogeneity. The model is used to perform ordinary pairwise meta-analysis comparing the different psychotherapy based on direct evidence from the clinical studies. Ranking probabilities will be calculated based on the joint distribution of the estimates of relative efficacy.^[29]^

Consistency will be addressed through the principle of node splitting by using a network meta-regression model. The purpose of node-splitting is to investigate if the relative effect of 2 psychotherapy based on direct comparisons is comparable with the same effect based on indirect comparisons. Statistically, the model is an extension of the NMA, which allows for a different relative effect between the 2 psychotherapy that are being split in head-to-head trials compared with all other trials. NMA will be implemented by the mvmeta software package in Stata (15.0; Stata Corporation, College Station, TX, USA Stata),^[30]^ If *P* value <.1 and I^2^ > 50%, it is considered that there is heterogeneity in the study, and sensitivity analysis or subgroup analysis will be performed to detect the source of heterogeneity. Funnel plot and Egger linear regression analysis will be used to assess publication bias. Using Review Manager (V.5.2.0) to analyze the risk of bias in the included studies, where the green, yellow, and red in the image represent low, unclear, and high risks, respectively.^[31,32]^

#### 2.5.3 Subgroup analysis

If statistical heterogeneity is evident, we will analyze the causes of heterogeneity, if there is enough data (such as differences between sexes, comparison between different countries, studies sponsored versus not sponsored by companies).

#### 2.5.4 Sensitivity analysis

We will use the exclusion method to conduct sensitivity analysis:

(1) exclude low-quality studies;
(2) exclude studies with comorbid physical or mental illnesses;
(3) exclude trials with missing data.

### 2.6 Quality of evidence

We will use Grading of Recommendations Assessment, Development and Evaluation (GRADE) framework to assess the quality of evidence for the primary outcomes.^[33,34]^ The quality of evidence is assessed as ‘high’, ‘moderate’, ‘low’ or ‘very low’. The following item will be evaluated: limitations, inconsistency, imprecision, indirectness, and publication bias.^[35]^

### 2.7 Summary of findings

A “summary of finding” table will be created for the major outcome. We will also add absolute and relative percentage changes to the “summary of finding”. For detailed information, see Table 3; we have listed partial summary of findings for the main comparison.

**Table 3:**
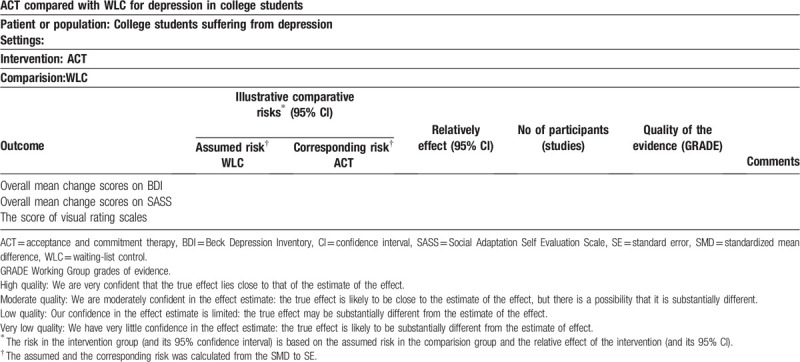
Summary of findings for the main comparison.

## 3 Result

### 3.1 Results of the search

**Figure 1:**
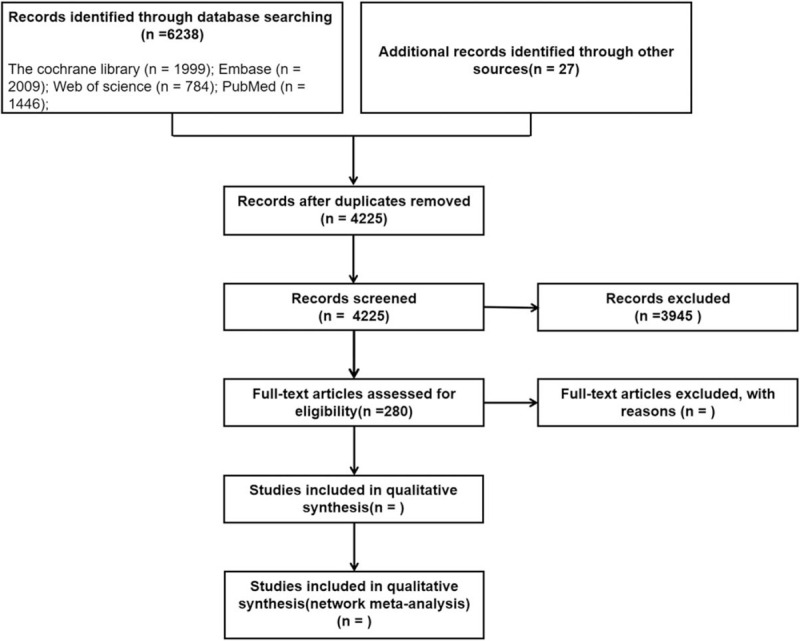
The flowchart of the screening process.

A total of 6238 records were obtained by searching the database and 27 records were obtained by other means. After removing duplicate records, there are 4225 records remaining. We excluded 3945 records through abstract and title, leaving 280 full-text articles. The document screening flowchart is shown in Figure 1.

### 3.2 Characteristic of included studies

In a preliminary trial, we included 8 studies. The average age of patients was 18 to 26, with a maximum sample size of 181 and a minimum sample size of 32. The research period ranges from one month to 12 months. For more detailed information, see Table 4.

**Table 4:**
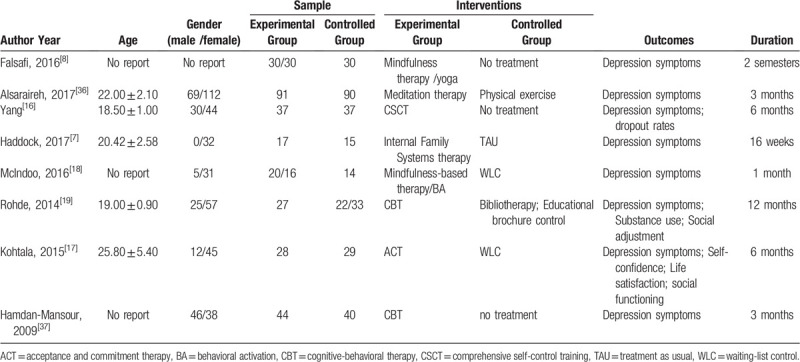
Basic characteristics of some of the included studies.

## 4 Discussion

At present, although some studies have evaluated the intervention effects of psychotherapy, there is no NMA to compare the therapeutic effects of different psychological interventions for college students. Therefore, this systematic review and NMA will summarize the direct comparison and indirect comparison evidence to evaluate different psychological interventions. We hope that this study will help guide clinical decision-making for psychotherapy to better treat depression in college students.

## Author contributions

**Conceptualization:** Xiu Zhang, Lin Wan.

**Data curation:** Xiu Zhang, Ming-Ming Niu, Pei-Fen Ma, Li Du, Lin Wan.

**Methodology:** Xiu Zhang, Lin Wan.

**Software:** Xiu Zhang, Ming-Ming Niu, Pei-Fen Ma, Li Du.

**Writing – original draft:** Xiu Zhang, Ming-Ming Niu, Lin Wan.

**Writing – review & editing:** Xiu Zhang, Lin Wan.

## References

[1] American Psychiatric Association. Diagnostic and Statistical Manual of Mental Disorders, Fifth Edition (DSM-5). Arlington: American Psychiatric Publishing; 2013.

[2] Yang FR Etiology, diagnosis and differential diagnosis of depression. Chin Med J 2005;40:53–5.

[3] Phillips MR Zhang J Shi Q Prevalence, treatment, and associated disability of mental disorders in four provinces in China during 2001-05: an epidemiological survey. Lancet 2009;373:2041–53. 19524780 10.1016/S0140-6736(09)60660-7

[4] Mayberg HS Lozano AM Voon V Deep brain stimulation for treatment resistant depression. Neuron 2005;45:651–60. 15748841 10.1016/j.neuron.2005.02.014

[5] World Health Organization. The global burden of disease: 2004 update. Geneva: World Health Organization; 2008.

[6] Cassano P Fava M Depression and public health: an overview. J Psychosom Res 2002;53:849–57. 12377293 10.1016/s0022-3999(02)00304-5

[7] Haddock SA Weiler LM Trump LJ The efficacy of internal family systems therapy in the treatment of depression among female college students: a pilot study. J Marital Fam Ther 2017;43:131–44. 27500908 10.1111/jmft.12184

[8] Falsafi N A randomized controlled trial of mindfulness versus yoga: effects on depression and/or anxiety in college students. J Am Psychiatr Nurses Assoc 2016;22:483–97. 27566622 10.1177/1078390316663307

[9] Bayram N Bilgel N The prevalence and socio-demographic correlations of depression, anxiety and stress among a group of university students. Soc Psychiatry Psychiatr Epidemiol 2008;43:667–72. 18398558 10.1007/s00127-008-0345-x

[10] Sartorius N Baghai TC Baldwin DS Antidepressant medications and other treatments of depressive disorders: a CINP Task Force report based on a review of evidence. Int J Neuropsychopharmacol 2007;10: Supply. 1: S1–207. 18096106 10.1017/S1461145707008255

[11] Qin B Zhou X Michael KD Psychotherapy for depression in children and adolescents: study protocol for a systematic review and network meta-analysis. BMJ Open 2015;5:e005918. 10.1136/bmjopen-2014-005918 PMC4330321 25681311

[12] Salanti G Higgins JP Ades AE Evaluation of networks of randomised trials. Stat Methods Med Res 2008;17:279–301. 17925316 10.1177/0962280207080643

[13] American Psychiatric Association. Diagnostic and statistical manual of mental disorders (DSM-III). 3rd Edition Washington, DC: American Psychiatric Association; 1980.

[14] American Psychiatric Association. Diagnostic and statistical manual of mental disorders (DSM-IV). 4th Edition Washington, DC: American Psychiatric Association; 1994.

[15] World Health Organization (WHO). The tenth revision of the international classification of diseases and related health problems (ICD-10). Geneva: World Health Organization; 1992.

[16] Yang X Zhao J Chen Y Comprehensive self-control training benefits depressed college students: A six-month randomized controlled intervention trial. J Affect Disord 2018;226:251–60. 29017069 10.1016/j.jad.2017.10.014

[17] Kohtala A Lappalainen R Savonen L A four-session acceptance and commitment therapy based intervention for depressive symptoms delivered by master degree level psychology students: a preliminary study. Behav Cogn Psychother 2015;43:360–73. 24229795 10.1017/S1352465813000969

[18] McIndoo CC File AA Preddy T Mindfulness-based therapy and behavioral activation: A randomized controlled trial with depressed college students. Behav Res Ther 2016;77:118–28. 26745622 10.1016/j.brat.2015.12.012

[19] Rohde P Stice E Shaw H Cognitive-behavioral group depression prevention compared to bibliotherapy and brochure control: nonsignificant effects in pilot effectiveness trial with college students. Behav Res Ther 2014;55:48–53. 24655464 10.1016/j.brat.2014.02.003 PMC3990276

[20] Beck A Steer R Garbin M Psychometric properties of the beck depression inventory: twenty-five years of evaluation. Clin Psychol Rev 1988;8:77–100. 1988.

[21] Radloff LS The CES-D scale: A self-report depression scale for research in the general population. Appl Psychol Meas 1997;1:385–401.

[22] Hamilton M A rating scale for depression. J Neurol Neurosurg Psychiat 1960;23:56–61. 14399272 10.1136/jnnp.23.1.56 PMC495331

[23] Bosc M Dubini A Polin V Development and validation of a social functioning scale, the Social Adaptation Self-Evaluation Scale. Eur Neuropsychopharmacol 1997;7: Suppl 1: 57–70. 9169311 10.1016/s0924-977x(97)00420-3

[24] Weissman MM Orvaschel H Padian N Children's symptom and social functioning self-report scales: comparison of mothers’ and children's reports. J Nerv Ment Dis 1980;168:736–40. 7452212 10.1097/00005053-198012000-00005

[25] Stice E Barrera M Jr Chassin L Prospective differential prediction of adolescent alcohol use and problem use: examining mechanisms of effect. J. Abnorm Psychol 1998;107:616–28. 10.1037//0021-843x.107.4.616 9830249

[26] Luo S Long Y Xiao W Risk of bias assessments and reporting quality of systematic reviews and randomized controlled trials examining acupuncture for depression: an overview and meta-epidemiology study. J Evid Based Med 2020;13:25–33. 32112515 10.1111/jebm.12372

[27] JPT CHH, Green S. Cochrane handbook for systematic reviews of interventions version 5.1. 0 [updated March 2011] The Cochrane Collaboration, 2011. http://www.cochrane-handbook.org [access date October 1 2011].

[28] Meulenkamp B Stacey D Fergusson D Protocol for treatment of Achilles tendon ruptures; a systematic review with network meta-analysis. Syst Rev 2018;7:247. 30580763 10.1186/s13643-018-0912-5 PMC6304227

[29] Baune BT Brignone M Larsen KG A network meta-analysis comparing effects of various antidepressant classes on the digit symbol substitution test (DSST) as a measure of cognitive dysfunction in patients with major depressive disorder. Int J Neuropsychopharmacol 2018;21:97–107. 29053849 10.1093/ijnp/pyx070 PMC5793828

[30] White IR Multivariate Random-effects meta-analysis. Stata J 2009;9:40–56.

[31] Ding N Zhang Z Zhang C What is the optimum time for initiation of early mobilization in mechanically ventilated patients? A network meta-analysis. PLoS One 2019;14:e0223151. 31589642 10.1371/journal.pone.0223151 PMC6779259

[32] Wang X Chen Y Yao L Reporting of declarations and conflicts of interest in WHO guidelines can be further improved. J Clin Epidemiol 2018;98:1–8. 29292204 10.1016/j.jclinepi.2017.12.021

[33] Norris SL Meerpohl JJ Akl EA The skills and experience of GRADE methodologists can be assessed with a simple tool. J Clin Epidemiol 2016;79:150–8. 27421684 10.1016/j.jclinepi.2016.07.001

[34] Puhan MA Schunemann HJ Murad MH A GRADE working group approach for rating the quality of treatment effect estimates from network meta-analysis. BMJ 2014;349:g5630. 25252733 10.1136/bmj.g5630

[35] Li XX Zheng Y Chen YL The reporting characteristics and methodological quality of Cochrane reviews about health policy research. Health Policy 2015;119:503–10. 25260911 10.1016/j.healthpol.2014.09.002

[36] Alsaraireh FA Aloush SM Mindfulness meditation versus physical exercise in the management of depression among nursing students. J Nurs Educ 2017;56:599–604. 28972629 10.3928/01484834-20170918-04

[37] Hamdan-Mansour AM Puskar K Bandak AG Effectiveness of cognitive -behavioral therapy on depressive symptomatology, stress and coping strategies among Jordanian university students. Issues Ment Health Nurs 2009;30:188–96. 19291496 10.1080/01612840802694577

